# Bone Fragment Co-transplantation Alongside Bone Marrow Aspirate Infusion Protects Kidney Transplant Recipients

**DOI:** 10.3389/fimmu.2021.630710

**Published:** 2021-02-11

**Authors:** Xianzhang Luo, Ji Zhang, Sijuan Zou, Xinqiang Wang, Gen Chen, Zhen Li, Kaiyan Li, Mengqing Wang, Zhishui Chen, Changshen Ming, Xiaohua Zhu, Nianqiao Gong

**Affiliations:** ^1^Key Laboratory of the National Health Commission, Institute of Organ Transplantation, Tongji Medical College, The Ministry of Education and Chinese Academy of Medical Sciences, Tongji Hospital, Huazhong University of Science and Technology, Wuhan, China; ^2^Key Laboratory for Biorheological Science and Technology of Ministry of Education, Chongqing University Cancer Hospital, Chongqing, China; ^3^Department of Nuclear Medicine, Tongji Medical College, Tongji Hospital, Huazhong University of Science and Technology, Wuhan, China; ^4^Department of Radiology, Tongji Medical College, Tongji Hospital, Huazhong University of Science and Technology, Wuhan, China; ^5^Department of Medical Ultrasound, Tongji Medical College, Tongji Hospital, Huazhong University of Science and Technology, Wuhan, China

**Keywords:** living kidney transplantation (LKT), bone fragment co-transplantation, bone marrow aspirate infusion, kidney protection, immune regulation

## Abstract

Integration of non-vascularized bone grafting and bone marrow aspirate infusion in transplantation may provide clinical benefit. Here we have incorporated bone fragment co-transplantation and bone marrow aspirate infusion (BF-BM) into living kidney transplantation (LKT). Twenty LKT recipients receiving bone fragments and bone marrow aspirates donated from their corresponding donors were enrolled into a retrospective study. A contemporaneous control group was formed of 38 out of 128 conventional LKT recipients, selected using propensity score matching by a 1:2 Greedy algorithm. Ultrasonography, contrast-enhanced ultrasonography (US/CEUS) and SPECT/CT showed that the co-transplanted bone fragments remained viable for 6 months, subsequently shrank, and finally degenerated 10 months post-transplantation. BF-BM resulted in earlier kidney recovery and more robust long-term kidney function. Throughout 5 years of follow-up, BF-BM had regulatory effects on dendritic cells (DCs), T helper (Th1/Th2) cells and regulatory T cells (Tregs). Both alloantigen-specific lymphocyte proliferation and panel reactive antibody levels were negative in all recipients with or without BF-BM. In addition, the BF-BM group experienced few complications during the 5-year follow-up (as did the donors)—this was not different from the controls. In conclusion, BF-BM is safe and benefits recipients by protecting the kidney and regulating the immune response.

## Introduction

Transplantation, the preferred treatment for end-stage organ failure, has been facilitated by the development of immunosuppressive agents that greatly reduce the incidence of rejection (1, 2). However, prolonged acceptance of transplanted organs requires the lifelong use of immunosuppressants. These can put the recipients and the grafts at risk of adverse effects, such as infection and acute/chronic injury that can become inexorable, limiting the lifespan of the transplanted organs and recipients (3–5). Therefore, discovery of new methods for graft protection and immunomodulation is essential.

A key lesson arises from the practice of composite allotransplantation (CAT), in which the predicted severe acute rejection does not occur (6–8). This identifies donor-derived bone co-transplantation as a new therapeutic option to protect the allograft (9–11). However, the vascularized bone transplants used in CAT are of limited feasibility in other settings such as kidney transplantation. First, procurement of intact vascular bone grafts remains challenging, especially in living donors. Second, the surgical site selected for a kidney transplant is commonly the iliac fossa, where there is no additional space for an intact bone. Finally, it can increase the risk of surgical complications. To date, no vascularized intact bone transplantations are performed.

One option, based on current clinical approaches used in orthotopics to cure bone defects, is to use non-vascularized bone fragment transplantation (12, 13). In living kidney transplantation (LKT), the classic flank approach of open donor nephrectomy with division of the ribs, provides a piece of bone currently seen as waste and discarded. Although laparoscopic techniques are currently popular in LKT, this approach via a mini-incision is still used in Europe, US, Asia and developing countries (14–19) and has similar benefits to a laparoscopic approach. An innovative approach would be to use fragments of the resected rib and implant them into an environment with an abundant blood supply as a non-vascularized bone co-transplant. To further mimic CAT, bone marrow is needed in addition to the bone grafts. Conveniently, bone marrow aspirate can be harvested via a biopsy technique currently in wide use by hematologists. Therefore, bone fragment co-transplantation with bone marrow aspirate infusion demonstrates feasibility in the current clinical environment.

Here we retrospectively studied a cohort of 20 LKT patients who received avascular bone fragment co-transplantation alongside bone marrow aspirate infusion, derived from their corresponding donors. These recipients were followed for 5 years. The dynamic evolution of the non-vascularized bone fragments after transplantation was tracked by review of imaging results and metabolic function evaluations. The data, including the status and complications of the donors and recipients, kidney function, chimerism and immune status readouts of recipients, were collected during a 5-year follow-up to assess clinical efficacy. Our work supports bone fragment co-transplantation alongside bone marrow aspirate infusion as a beneficial clinical option in kidney transplantation.

## Materials and Methods

### Patients

From May 2011 to May 2013, 149 LKTs were performed in our institution. The selection criteria for donor and recipient complied with the Regulations of Organ Transplantation of the People's Republic of China, as previously described (20). Among the 149 pairs, 21 recruited pairs were fully informed of the procedure and possible related complications/advantages for both donor (kidney procurement, 11th rib resection, bone marrow harvesting) and recipient (kidney transplantation, bone fragments implantation, bone marrow aspirate infusion). After giving written informed consent, the 21 pairs underwent LKT and bone fragment co-transplantation plus bone marrow aspirate infusion (BF-BM), with approval from the institutional review board at Tongji Hospital, Tongji Medical College, Huazhong University of Science and Technology (ethical approval no. 201302009). All 149 pairs were followed beyond 5 years. To evaluate the impacts of BF-BM on the donors and recipients, the 128 pairs not receiving BF-BM were treated as controls. A retrospective chart review was performed, and a propensity score matching method was used to select subjects from the 21 BF-BM pairs and 129 control pairs for further analysis, in order to reduce confounding effects of baseline age, HLA-mismatches in MHC-I and MHC-II, donated kidney glomerular filtration rate (GFR), warm ischemia time and cold ischemia time. Finally, 20 pairs were selected as the BF-BM group and 38 pairs as the control group, using a 1:2 Greedy algorithm. This retrospective study was approved by the institutional review board at the National Ministry of Health.

### Bone Fragment Co-transplantation and Bone Marrow Aspirate Infusion

A 10 cm-length trans-11th rib flank incision was used for donor surgery. From the donor, the 11th rib was harvested, cut into 10–15 small fragments, and preserved in 4°C histidine-tryptophan-ketoglutarate solution. After kidney procurement, bone marrow puncture was performed under anesthesia at the bilateral iliac crests, and 100 ml bone marrow was harvested, filtered and prepared as a bone marrow aspirate suspension, which was stored in blood storage solution until infusion.

The recipient underwent a standard kidney transplantation procedure. After grafting, the abdominal muscle near the kidney surface was bluntly dissected to form a compartment that provided an environment with a blood supply and space where the bone fragments were implanted. After surgery, the recipient was given the bone marrow aspirate infusion through the peripheral vein, which proceeded for 2 h under stable vital signs.

### Management and Follow-Up of Donors and Recipients

No induction therapy was given to all the recipients. After transplantation, all the recipients received maintenance immunosuppressive therapy with the protocol as tacrolimus (Tac)+ mycophenolic acid (MPA)+prednisone.

Any complications related to the surgery and bone marrow harvest in the living donors were documented.

The recipients were monitored daily during the 1st week and every 2–3 days during the 2nd week. Delayed graft function (DGF), defined as the need for dialysis at least once within the 1st week post-transplantation (21), and primary non-function (PNF) were recorded as key kidney damage parameters to evaluate recovery of kidney function. Post discharge follow-ups were conducted weekly for the first trimester, fortnightly for the next 3 months and monthly thereafter and complications were documented. The 5-year follow-up data were collected retrospectively.

Kidney allografts and bone fragments were scanned by ultrasonography, contrast-enhanced ultrasonography and hybrid SPECT-CT at different stages, including 1 month (ranging from 10 days to 4 weeks), 3 months (ranging from 2 to 4 months), 6 months (ranging from 5 to 7 months), 10 months (ranging from 8 to 12 months), and 15 months (ranging from 13 to 18 months) post-transplantation.

Renal function was tested at each follow-up time-point. The serum creatinine level and eGFR, calculated from the adapted Modification of Diet in Renal Disease (MDRD) equation (22), were documented. At 6 months and 1, 3, and 5 years post-transplantation, PBMCs and sera of the recipients were collected to detect their immune reactivity. Tac trough levels were examined at each follow-up and were adjusted based on the clinical status.

### Ultrasonography and Contrast-Enhanced Ultrasonography (US/CEUS)

US/CEUS was performed using a scanner (GE LOGIQ19) equipped with TIC software with use of the superficial transducers (9L). First, bone fragments were assessed for their presence or absence by acoustic shadowing and hyperechoic values around the kidney graft. Then dynamic real-time CEUS was performed in conformity, with a 10 ml syringe containing 5 ml of saline solution and 1 ml of microbubble, of which 2.4 ml was bolus-administered. If the peri-fragment showed enhancement, the following parameters were then recorded: time to enhancement (TTE), time to peak (TTP) and the maximum blood flow of the peri-bone area (MBF).

### Bone Hybrid SPECT-CT Scanning

SPECT-CT images of bone fragments were performed 3 h after intravenous administration of 740 MBq (20 mCi) of 99mTc-methylene diphosphonate (MDP). SPECT-CT scans were obtained using a dual head variable angle SPECT equipped with a 16-slice helical CT scanner (Discovery 670 SPECT/CT, GE Healthcare Technologies, USA). Tracer uptake in the allogeneic bone fragments (B) and normal soft tissue on the contra-lateral side (N) were assessed by semi-quantifying volumes of interest (VOI) of B and N, manually delineated in transversal SPECT-CT images using CT as the anatomical map. The radioactivity ratio was calculated between the Max VOI counts of B to N (BMax/NMax).

### Recipients' Immune Status

Peripheral blood mononuclear cells (PBMCs) were obtained from recipients by density gradient centrifugation using LTS-1077 (density = 1.077 g/ml; TBD, Tianjin Haoyang Biological Manufacture Co. Ltd., Tianjin, China), and preserved at −80°C using CELLSAVING (New Cell & Molecular Biotech, Suzhou, China). The recipients' PBMCs were thawed and washed in PBS once and incubated with anti-human CD3, CD4, CD8, CD11c, CD16, CD25, CD56, CD68, CD80, CD86, CD206, and HLA-DR antibodies (Biolegend; San Diego, CA, USA) in the dark at 4°C for 30 min. For intracellular staining, cells were fixed and permeabilized with Fixation & Permeabilization Buffer (eBiosciences; Thermo Fisher Scientific, Inc., Waltham, MA, USA) for 30 min, then stained with anti-human Foxp3, IFN-γ, IL-4, IL-17A antibodies (Biolegend; San Diego, CA, USA) in 1 ml PBS. The proportions of DC (CD11c+, HLA-DR+), Th1 (CD4+, IFN-γ+), Th2 (CD4+, IL-4+), CTL (CD3+, CD4+, CD8+), Th17 (CD4+, IL-17A+), Treg (CD4+, CD25+, Foxp3+), MΦ (CD68+), NK (CD3-, CD16+, CD56+), and NKT (CD3+, CD56+) in recipient PBMC were analyzed in duplicate.

Recipient sera were thawed and assessed for levels of serum cytokines including IL-2, TNF-α, IFN-γ, IL-17F, IL-4, IL-6, IL-9, IL-10, and IL-13 by flow cytometry using the LEGENDplex™ Multi-Analyte Flow Assay Kit (Biolegend; San Diego, CA, USA). The assays were repeated in all samples in duplicate.

The recipients' PBMCs were stained with CFSE (Biolegend) using the manufacturer's protocol and co-cultured with donor PBMC at a ratio of 5:1 in a 96-well round-bottomed plate, in triplicate. Proliferation was determined using flow cytometric analysis of CFSE dilution. Recipient PBMC in the absence of donor PBMC were used as the negative control. Recipient PBMC stimulated with anti-CD3 and CD28 antibodies (eBioscience/Thermo Fisher Scientific; San Diego, CA, USA) were used as the positive control.

To detect levels of humoral immunity, panel reactive antibodies (PRA) were detected by LABScan™ 100 flow cytometry using the LABScreen-PRA Kit (One Lambda, Inc., Canoga Park, CA, USA). PRA <10% was considered negative.

### Statistics

The control group was used as comparator for evaluating the safety and efficacy of the procedure. Continuous variables were reported as means and standard deviations. The mean, median, range, and frequency were used as descriptive statistics. Continuous variables, including renal function, eGFR, immune responses and cytokines between the two groups were compared using the Student's *t*-test or the Mann–Whitney *U*-test as appropriate. Categorical variables were compared by means of Fisher's exact test. All analyses were conducted using SAS 9.4 software (SAS Institute Inc., Cary, NC, USA).

## Results

### Demographics

The baseline characteristics of the 58 renal allograft recipients and their donors are presented in Table 1. The recipient age, HLA compatibility, warm ischemia time, gender, basal panel reactive antibody, time undergoing dialysis, cold ischemia time and donor eGFR were all comparable between the two groups.

**Table 1:** Recipient and Donor Demographics.

	**Control (*n* = 38)**	**BF-BM (*n* = 20)**	***P*-value**
Recipient age (years)	26.1 ± 5.3	26.0 ± 6.3	0.75
Gender			0.54
Male (n)	27	13	
Female (n)	11	7	
Pretransplant dialysis type			0.32
Hemodialysis	38	19	
eritoneal dialysis	0	1	
Prior dialysis time (mon)	16.5 ± 18.0	14.9 ± 16.8	0.49
Panel reactive antibody (%)			
MHC-I	3.1 ± 2.7	3.1 ± 3.4	0.98
MHC-II	2.2 ± 2.2	2.2 ± 2.9	0.87
Mismatch of six HLA antigens	1.3 ± 0.9	1.4 ± 0.9	0.26
Warm ischemia time (minutes)	1.4 ± 1.0	1.7 ± 0.7	0.25
Cold ischemia time (minutes)	165.7 ± 38.9	163.4 ± 29.2	0.80
Donor age (years)	48.5 ± 7.2	48.9 ± 8.0	0.65
Donor gender			0.54
Male (n)	11	6	
Female (n)	27	14	
Kidney GFR before donation	45.5 ± 5.5	45.8 ± 6.1	0.81

*MHC, major histocompatibility complex; GFR, glomerular filtration rate (detected before donation)*.

### Complications in Donors After Rib Resection and Bone Marrow Harvest

The perioperative and long-term complications, including operation time, pleural entry, postoperative average narcotic use, wound infection, chest infection, incisional hernia, mortality, chronic wound pain up to 1 year post-surgery, painful puncture points, and changes in leukocytes and hemoglobin from pre-operation to POD 5, are documented in Table 2. No significant differences were found between controls and the BF-BM group.

**Table 2:** Complications of the Donors.

	**Control (*n* = 38)**	**BF-BM (*n* = 20)**	***P*-value**
Operation time (minutes)	154 ± 8.4	155 ± 8	0.26
Pleural entry	1	1	1
Blood transfusion	0	0	–
**Postoperative**			
Average narcotic use (hr)	33.6 ± 2.4	33.2 ± 1.8	0.37
Wound infection	0	0	–
Chest infection	0	0	–
Incisional hernia	0	0	–
Mortality	0	0	–
Chronic wound pain (1 y post-surgery)	3	1	1
Puncture point painful	2	1	1
**White blood cells (10∧9)**			
Pre-operation	6.1 ± 0.6	6.2 ± 0.8	0.19
POD 5	6.0 ± 0.6	6.2 ± 1.0	0.17
**Hemoglobin (g/L)**			
Pre-operation	122.6 ± 6.8	122.3 ± 6.4	0.10
POD 5	118.7 ± 5.5	117.3 ± 6.0	0.14
**Albumin (g/L)**			
Pre-operation	45.8 ± 2.0	46.1 ± 2.5	0.45
POD 5	43.8 ± 2.6	43.7 ± 2.8	0.11

*POD, post operation day*.

### Detection of Co-transplanted Bone Grafts Up to 10 Months

**Figure 1:**
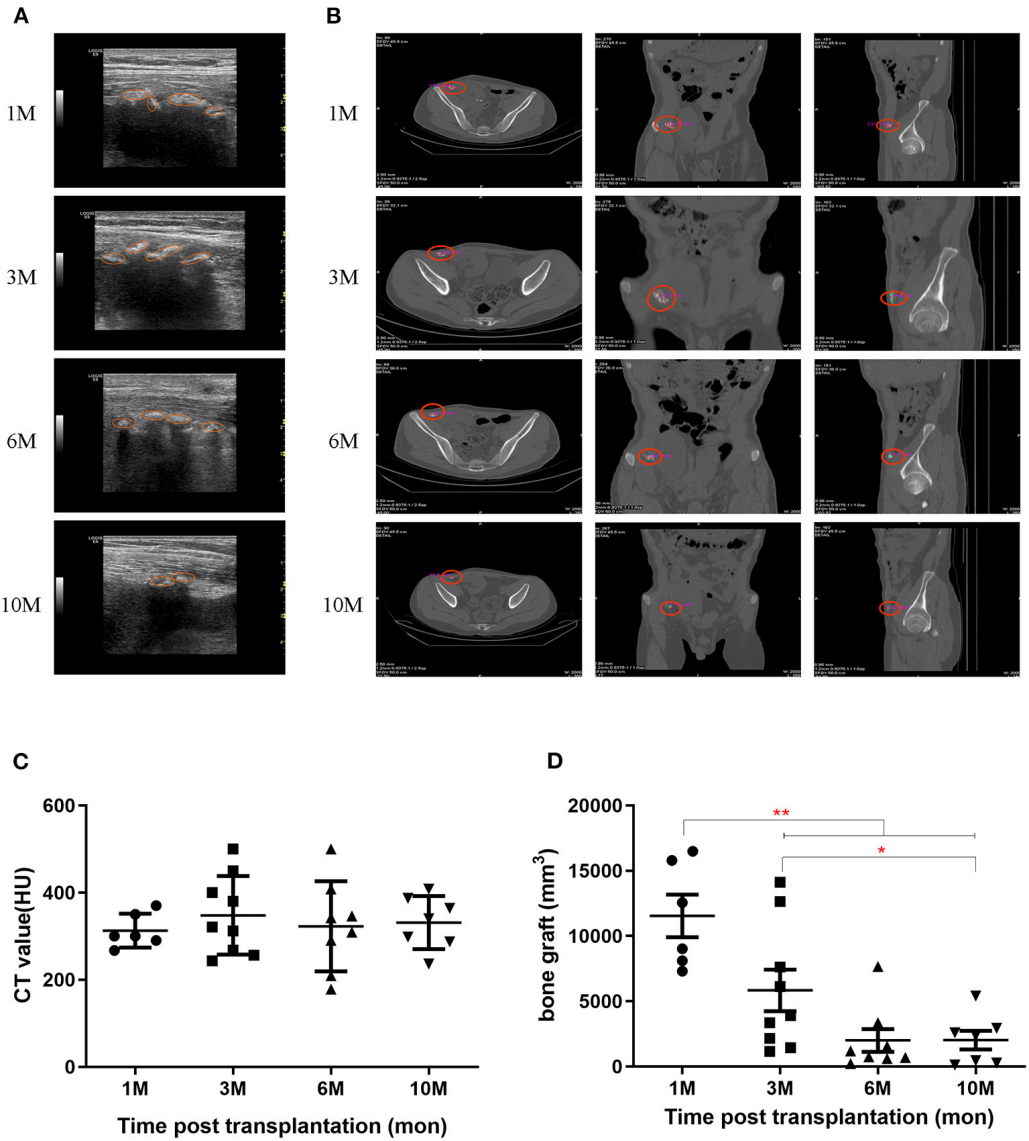
Persistence of bone grafts post-transplantation. **(A)** Bone grafts were detected by ultrasound at 1, 3, 6, and 10 months post-transplantation. The orange circles indicate the ranges for each bone graft. **(B)** Bone grafts were detected by CT scan at 1, 3, 6, and 10 months post-transplantation. **(C)** Bone graft CT values were detected by CT scan. **(D)** Bone graft volumes were detected by CT scan. *p < 0.05; **p < 0.01.

The transplanted bone fragments were observed for bone-specific acoustic shadowing and hyperechoic values around the kidney allograft using gray-scale ultrasonography. During the 6 months post-transplantation, the acoustic shadowing maintained high hyperechoic values. However, the shadow and hyperechoic values subsequently decreased and the bone allografts became faintly distinguishable beyond 1 year, compared to the adjacent mesenchymal tissue (Figure 1A). Bone fragments were also monitored by CT scanning (Figure 1B). CT values at each follow-up were quantified as 313 ± 39, 348 ± 90, 323 ± 103, and 331 ± 61 HU at 1, 3, 6, and 10 months, respectively (Figure 1C). The volumes of the bone grafts measured by CT scan at each follow-up were quantified as 11,545 ± 3,991, 5,839 ± 4,780, 2,004 ± 2,481, and 2,027 ± 1,960 mm^3^ at 1, 3, 6, and 10 months, respectively (Figure 1D).

### Reconstructed Blood Supply and Persistence for 10 Months of Co-transplanted Bone Grafts

**Figure 2:**
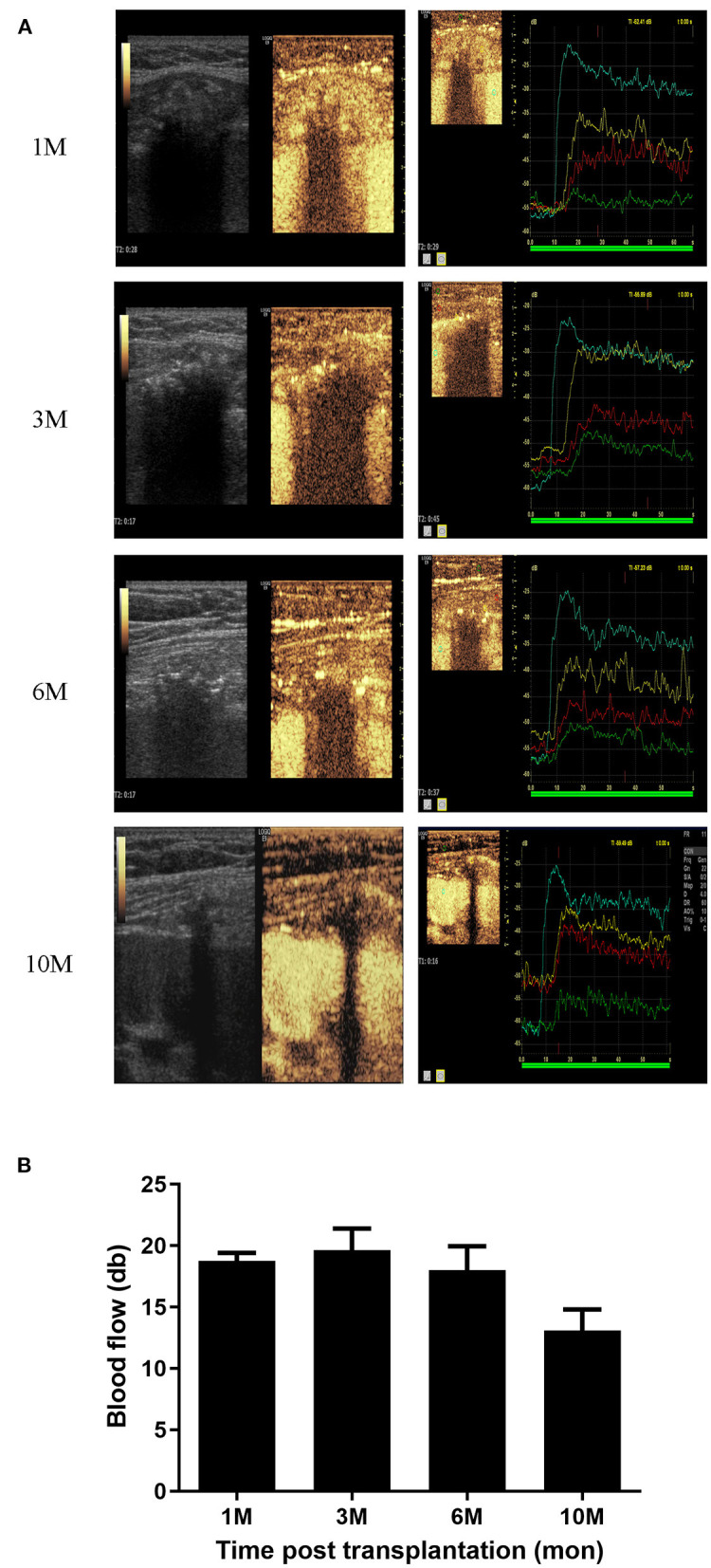
Blood supply analysis of bone grafts. **(A)** CEUS photography of the bone grafts at 1, 3, 6, and 10 months post-transplantation. **(B)** Blood supply of bone grafts represented by MBF values at 1, 3, 6, and 10 months post-transplantation.

CEUS describes blood flow by time to enhancement (TTE), time to peak (TTP) and maximum blood flow in the peri-bone area (MBF). A total of 31 examinations were performed in recipients at different time-points (1, 3, 6, and 10 months). The bone grafts exhibited specific features based on the parameters TTE, TTP, and MBF, which we compared with the kidney and nearby muscle (Supplementary Figures 1A–C). The blood supply of the bone grafts was found to fluent soon after transplantation (10 days) and the MBF values were maintained for 6 months. However, MBF values decreased from 6 months post-transplantation and became faint 10 months post-transplantation (Figures 2A,B).

### Co-Transplanted Bone Grafts Maintain Metabolic Activity for 1 Year

**Figure 3:**
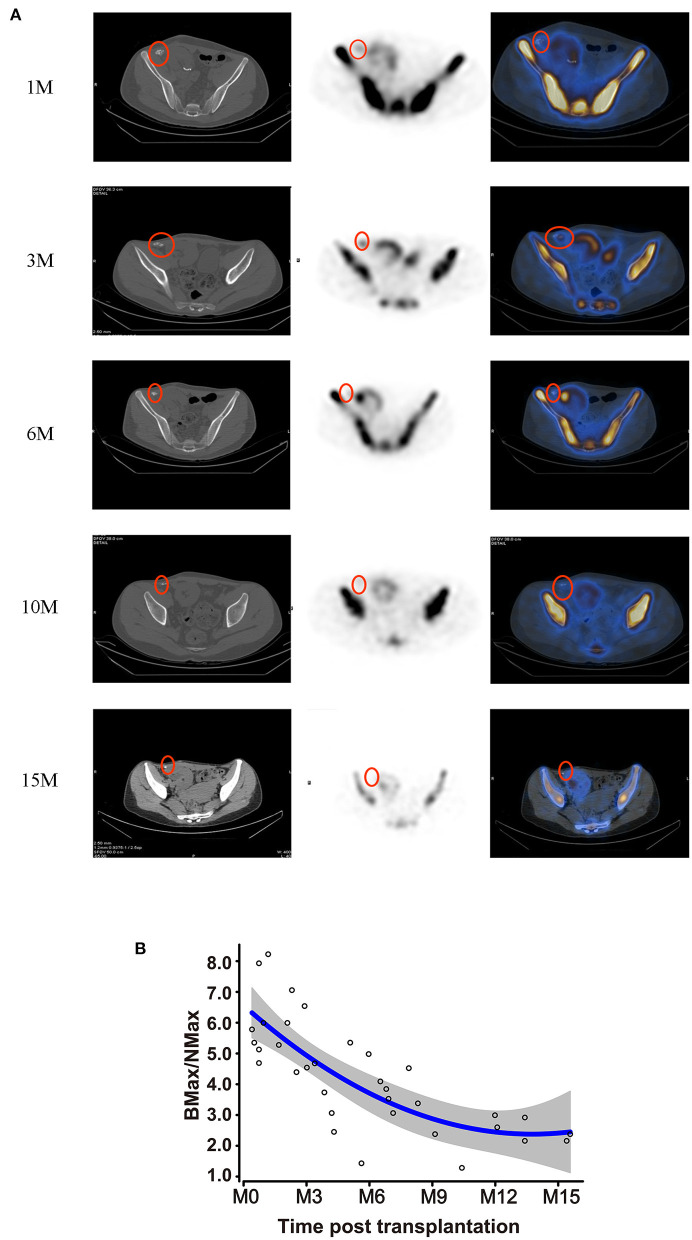
Metabolic activity of bone grafts. **(A)** Hybrid SPECT/CT photography of the bone grafts at 1, 3, 6, 10, and 15 months post-transplantation. **(B)** Metabolic viability of the bone grafts, represented by BMax/NMax values, showing alterations according to the time-point post-transplantation.

Bone hybrid SPECT-CT imaging with 99mTc-MDP detected the distribution of osteoblastic activity to evaluate the function of bone grafts. Recipients received 41 ECT examinations at different time-points (1, 3, 6, 10, and 15 months). The radiotracer uptake of the bone allografts is shown in Figure 3A, and was stronger than normal in soft tissue on the contra-lateral side, but lower than the recipients' own iliac crest. The Max VOI count of bone to normal soft tissue (BMax/NMax) was used to evaluate metabolic function. BMax/NMax values at each follow-up are plotted in Figure 3B. Metabolic function was maintained during the first 6 months; subsequently it gradually decreased but still maintained a residual level at 10 months. Interestingly, though still decreasing, metabolic activity remained detectable at 15 months.

### BF-BM Promotes Early Kidney Recovery and Long-Term Kidney Function in Recipients

**Figure 4:**
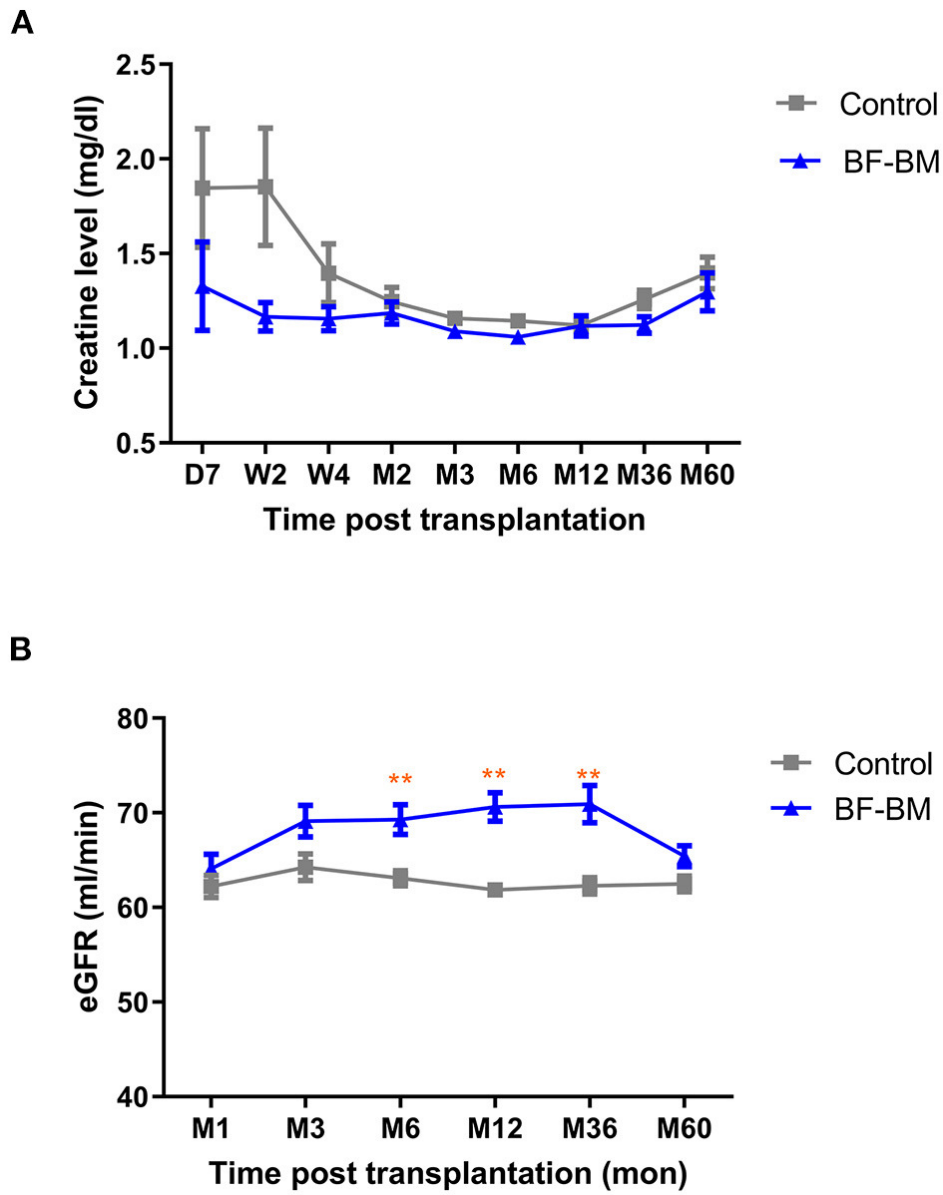
Kidney function post-transplantation. **(A)** Creatinine levels of the recipients throughout the 5-year follow-up. **(B)** eGFR throughout the 5-year follow-up. **, *p* < 0.01.

All recipients achieved immediate kidney function with no occurrence of DGF/PNF. No graft/recipient losses occurred during the whole follow-up period. Throughout the 5-year follow-up, absolute serum creatinine levels in the BF-BM group appeared lower than in the control group, especially during the first 4 weeks, although the difference was not statistically significant (Figure 4A). The eGFR, which incorporates factors including age and sex to further evaluate kidney function using the MDRD equation, was higher in the BF-BM group than that in controls post-transplantation, with statistically significant differences at timepoints of 6, 12, and 36 months post-transplantation (Figure 4B).

### BF-BM Has No Impact on Chimerism

One year post-transplantation, all the BF-BM recipients underwent STR detection of CD3+ cells in peripheral blood. All the CD3+ cells belonged to the recipient. Therefore, there was no evidence of T cell chimerism.

### BF-BM Exerts Regulatory Effects on Immune Reactivity

**Figure 5:**
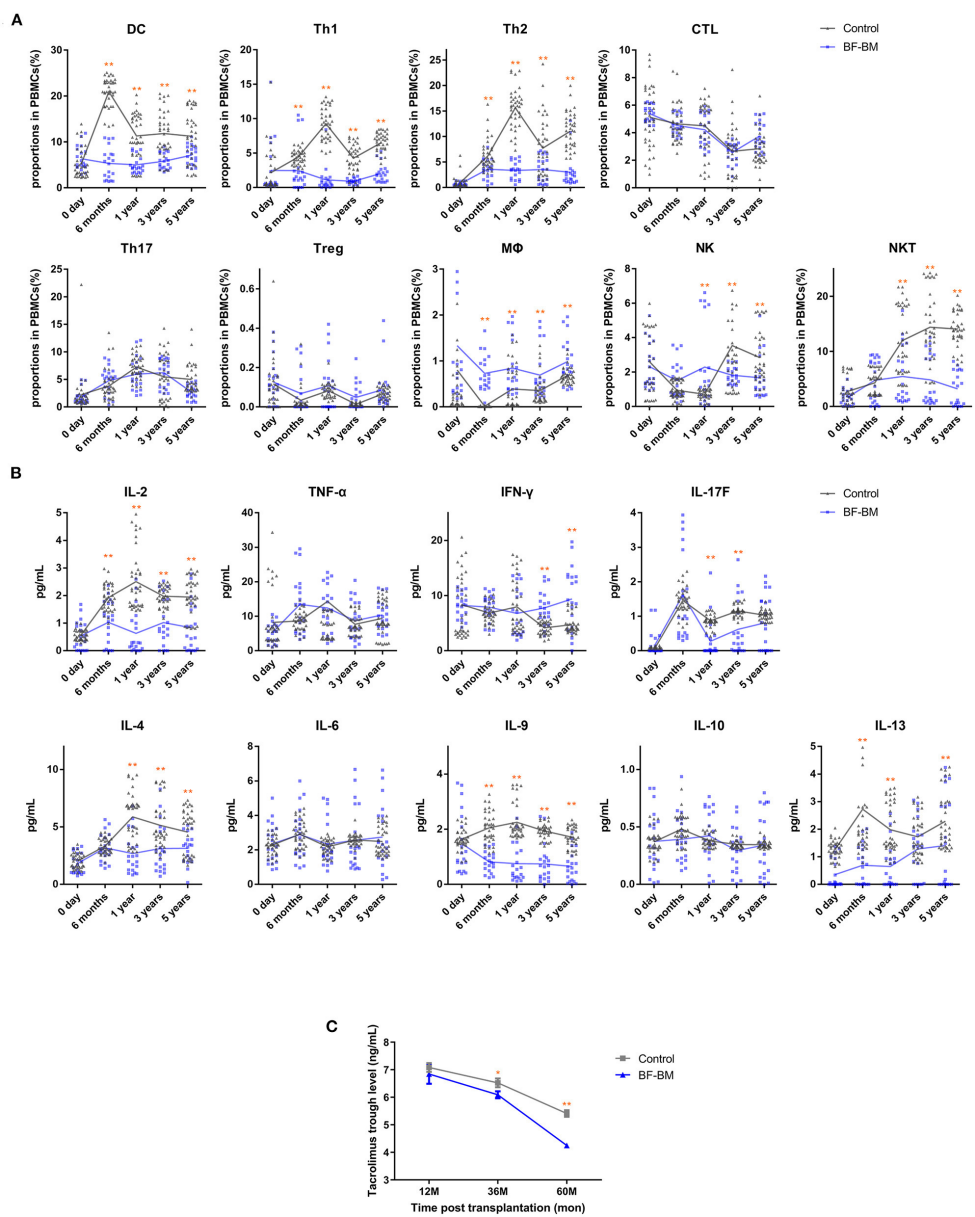
Recipient immune status after transplantation. **(A)** Proportions of DC, Th1, Th2, CTL, Th17, Treg, MΦ, NK, and NKT in PBMCs at day 0, 6 months, 1 year, 3 years, and 5 years post-transplantation. **(B)** Serum levels of IL-2, IL-6, TNF-α, IFN-γ, IL-17F, IL-4, IL-6, IL-9, IL-10, and IL-13 at the same time points. Assays were performed in duplicate and each dot represents one replicate. **(C)** Tacrolimus trough levels at 1, 3, and 5 years post-transplantation. *, *p* < 0.05; **, *p* < 0.01.

Recipient immune status was evaluated by assessment of PBMC profile, serum cytokines (Figures 5A,B), alloantigen-specific lymphocyte proliferation against the corresponding donor, and PRA levels. PBMC profiles showed no differences between the BF-BM and control groups prior to transplantation. However, the proportions of DCs, Th1 and Th2 were significantly decreased in the BF-BM group compared to controls, although no differences were identified for CTL or Th17. Although not statistically significant, there was a slight trend toward higher Tregs in the BF-BM group throughout the 5-year follow-up period. With regards to innate immune cells, trends of higher MΦ, lower NKT and variable NK cell levels were observed. The data suggest that a beneficial regulatory effect on acquired immunity was induced in the BF-BM group compared to no intervention. Regarding cytokine profiles in serum, significant changes were identified in the BF-BM group throughout the follow-up. For Th1 cytokines, TNF-α was not different but INF-γ was higher from 3-year in the BF-BM group. IL-17F was lower in the BF-BM group throughout the follow-up. Th2 cytokines, including IL-4, IL-9 and IL-13, were lower in the BF-BM group. These findings suggest that bone fragment co-transplantation with bone marrow aspirate infusion induces regulatory effects in the recipients.

At 1, 3, and 5 years post-transplantation, co-culture of recipient PBMCs with donor mitomycin-treated PBMCs resulted in very low proliferation in both groups, indicating no alloantigen-specific T cell activation. In addition, PRA levels were negative in both groups, suggesting there was no alloantigen-specific B cell activation.

Based on the stable kidney function and clinical status of the recipients, use of tacrolimus was individually tailored for trough levels. Tacrolimus exposure maintained relatively low trough levels and the value in the BF-BM group was lower than that in the control group (Figure 5C). Meanwhile, 3 episodes of rejection were clinically diagnosed, of which one was in the BF-BM group and two in the control group. All the rejections were rapidly reversed by methylprednisolone bolus treatment. Interestingly, using less immunosuppressant benefited the recipients since there was a lower rate of opportunistic infection (Table 3).

**Table 3:** Complications of the recipients post-transplantation.

	**Control (*n* = 38)**	**BF-BM (*n* = 20)**
DGF	0	0
Acute rejection	2	1
Biopsy-confirmed	1	0
Corticosteroid-resistant	0	0
Infections	14	5
Pneumonia	9	2
Upper respiratory tract infection	2	3
Herpes zoster	1	0
Urinary tract infection	1	0
Tuberculosis	1	0

### Recipient Safety and Survival

All patients' postoperative course was uneventful. All recipients survived, and no surgical complications, life-threatening complications or GVHD occurred during the 5-year follow-up.

## Discussion

Although laparoscopic donor nephrectomy has been adopted as the prevailing method (23), open surgery is still undertaken worldwide due to considerations that include its lower operative and warm ischemia times, lower risk of intra-abdominal insufflation and reduced cost. Using a flank incision with 11th rib resection, which provides better dissection of the renal pedicles (especially for high-residing kidneys) (14–19), donor ribs could be re-used in this study. Careful monitoring of donors showed that combined kidney and bone marrow donation resulted in no more complications than currently reported, demonstrating the acceptability and safety of the procedure.

We observed the evolution of the co-transplanted bone fragments by ultrasound and radiology. After transplantation, ultrasound confirmed the survival of bone grafts beside the kidney grafts and CT scans accurately identified their positions and quantified CT values and volumes of each bone graft. Both ultrasound and CT revealed that bone grafts maintained for 6 months post-transplantation. Subsequently, co-transplanted bone tissue shrank.

The blood supply of the avascular bone grafts was further assessed by CEUS, a well-established technique that provides highly accurate information about the vasculature with enhancement pattern (24, 25) and evaluates the viability of osteocutaneous free flaps after transplantation (26–28). In our case series, the blood supply of the bone grafts was reconstituted rapidly and was maintained for 6 months. However, blood supply decreased from 6 months post-transplantation and was faint 10 months post-transplantation. These findings suggest that bone grafts survive for 10 months post-transplantation. Notably, although the bone grafts had lower peak blood flow and later TTP than soft tissue (27, 28), they had higher peak flow and earlier TTP than nearby muscle at the early post-operative stage. This may be partly because the free bone microenvironment had a wider contact area to the soft tissue, and accordingly the blood supply was altered in comparison to an intact bone.

The functional metabolic activity of the avascular bone grafts was then examined. Radionuclide bone scanning has been widely accepted for such assessment of bone grafts (12, 29–31). Hybrid SPECT-CT allows for fusion of the functional SPECT frames with the CT anatomical images and thus can provide a precise anatomical location for any abnormal uptake (30) - this is superior to planar bone scintigraphy (32). We found that the bone grafts had adequate metabolic function during the first 6 months, which decreased thereafter. Interestingly, in contrast to the findings from CEUS, 99mTc-labeled MDP uptake remained detectable even beyond a year post-transplantation, although the volume of the bone grafts had become minimal. The degeneration of bone grafts could be partly explained by allo-immune reactivity and the heterotopic environment which might cause bone fibrosis. We also found that radiotracer uptake of the bone allograft was lower than in the recipients' own iliac crest, indicating that the function of the bone allografts was reconstituted but did not fully recover.

Overall, the dynamic evolution of the co-transplanted bone fragments can be summarized as 1: fragments regain a blood supply soon after transplantation; 2: the structure and function of the bone grafts remain satisfactory for 6 months and decrease thereafter; 3: bone grafts degenerate 10 months post-transplantation.

Clearly it is important to evaluate the outcome of the donor bone marrow cell transplants in recipients. One week after transplantation, there was no change in the proportion of CD34+ cells in PBMC (data not shown), which may be explained by the low numbers of stem cells within bone marrow cells. The mechanism behind the benefits of bone marrow may be related to stem cells. One year after transplantation, chimerism detection by STR failed to show evidence for donor-derived cells in the recipient circulation.

The follow-up of recipients' kidney function throughout the 5-year period showed that all the recipients gained immediate kidney function without DGF/PNF. The sCr levels in the BF-BM group remained lower than those in controls, especially during the first 4 weeks. The eGFR was consistently higher in the BF-BM group than in controls. These findings suggest that BF-BM protects the kidneys, enabling early kidney recovery and long-term kidney function.

Although basic research has shown that free bone co-transplantation combined with conventional immunosuppression can induce immunologic tolerance and extend allograft survival (33–37), in this study tolerance was not achieved and was not the target of therapy. However, regulatory effects of BF-BM on the immune system could still be identified. Prior to transplantation, there was no difference in PBMC profile between BF-BM and control group. After BF-BM, decreased proportions of DCs, Th1 and Th2, but not CTL or Th17, were observed throughout the 5-year follow-up. Intriguingly, the proportion of Treg showed a modest trend toward an increase in BF-BM, without reaching statistical significance. BF-BM had very subtle effects on innate immunity, reflected by divergent trends including higher MΦ, lower NKT and variable NK proportions. Cytokine profiles in serum indicated that IL-2, IL-17F, and Th2 cytokine (IL-4, IL-9, and IL-13) levels were decreased by BF-BM throughout follow-up, and were generally consistent with the PBMC profiling results. Allo-antigen specific T cell proliferation, assessed by the co-culture of recipient PBMC with donor mitomycin-treated PBMC, and B cell activity (reflected by PRA levels) were all negative, suggesting low immunological reactivity post LKT. Taken together, our data suggest regulatory effects of BF-BM on DCs, Th1/Th2 cells and Tregs which were benefit to the immune-protection on the transplanted kidney and bone fragments.

The kidney protection and immune regulatory effects are based on the features of the transplanted bone fragments and infused bone marrow aspirate. After transplantation, the donor kidney and its nearby bone fragments, as damaged tissues, may secrete chemokines such as SDF-1α (38–40) to induce the mesenchymal stromal cells (MSCs) in the donor's bone marrow migrating into the donor kidneys (41) to exert immunomodulatory properties (42). On the other hand, the hematopoietic stem cells (HSCs) in donors' bone marrow may home to the bone fragments/recipients' bone-marrow guided by adhesion molecules (VLA4, VLA5, LFA1, selectins, et al.) and lodge in the niche by SCF, CXCL12, osteopontin (43–46) resulting in deletion or anergy of donor-reactive T cells (42, 47–51). To date, the comprehensive cellular and molecular mechanisms remain unclear and await further investigation.

Direct clinical evidence for low immunological reactivity status was provided by the relatively low tacrolimus exposure in individually tailored regimens and rare and mild rejection episodes. Less immunosuppressant exposure is beneficial, by decreasing the rate of opportunistic infection and tacrolimus nephrotoxicity and results in good safety without additional complications (for both the kidney grafts and recipients). Thus, taken together, BF-BM has the advantages of modulating immune reactivity, decreasing adverse effects and decreasing economic burden.

**Figure 6:**
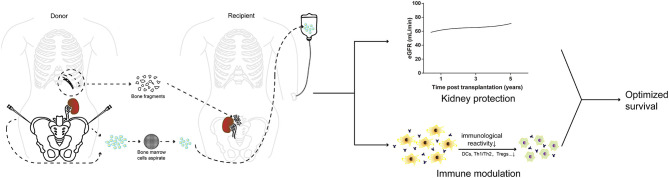
Bone fragment co-transplantation alongside bone marrow aspirate infusion protects kidney transplant recipients.

Our reported study has some limitations, including its retrospective exploratory nature, the relatively small number of cases, lack of bone fragment biopsy data and the fact that US/CEUS and SPECT/CT were not performed at every time-point. However, it demonstrated clear effects of BF-BM on kidney protection and immune regulation, providing a novel and feasible therapeutic option for LKT recipients (as illustrated in Figure 6). This approach could be easily extended to deceased-donor transplantation, which would benefit more transplant patients. Further development of this strategy is warranted and a prospective randomized controlled clinical trial is required.

## Data Availability Statement

The raw data supporting the conclusions of this article will be made available by the authors, without undue reservation.

## Ethics Statement

The studies involving human participants were reviewed and approved by Medical Ethics Committee of Tongji Hospital, Tongji Medical College, Huazhong University of Science and Technology. The patients/participants provided their written informed consent to participate in this study. Written informed consent was obtained from the individual(s) for the publication of any potentially identifiable images or data included in this article.

## Author Contributions

NG and XZ: guarantors of integrity of the entire study. XL, SZ, XW, GC, XZ, and NG: literature research. XL, XW, MW, JZ, and NG: clinical studies. XL, SZ, and XW: statistical analysis. All authors: study concepts, study design, data acquisition, data analysis, interpretation, manuscript drafting, manuscript revision for important intellectual content, approval of the final version of the submitted manuscript, agreement to ensure any questions related to the work are appropriately resolved, and manuscript editing.

## Conflict of Interest

The authors declare that the research was conducted in the absence of any commercial or financial relationships that could be construed as a potential conflict of interest.
