# Supplementary material for: Bone Fragment Co-transplantation Alongside Bone Marrow Aspirate Infusion Protects Kidney Transplant Recipients
Source: Front Immunol. 2021 Feb 11;12:630710. doi: 10.3389/fimmu.2021.630710 (PMC7904687; doi:10.3389/fimmu.2021.630710)
Supplement: Supplementary file 1 [file Data_Sheet_1.PDF]

Supplementary Figure 1. Comparison of TTE, TTP and MBF between the control, BF-BM and BF groups throughout the year post-transplantation. A, TTE; B. TTP; C, MBF. Renal grafts, bone grafts and muscles are shown at 1, 3, 6 and 10 months post-transplantation.

Figure 1

Figure 1A

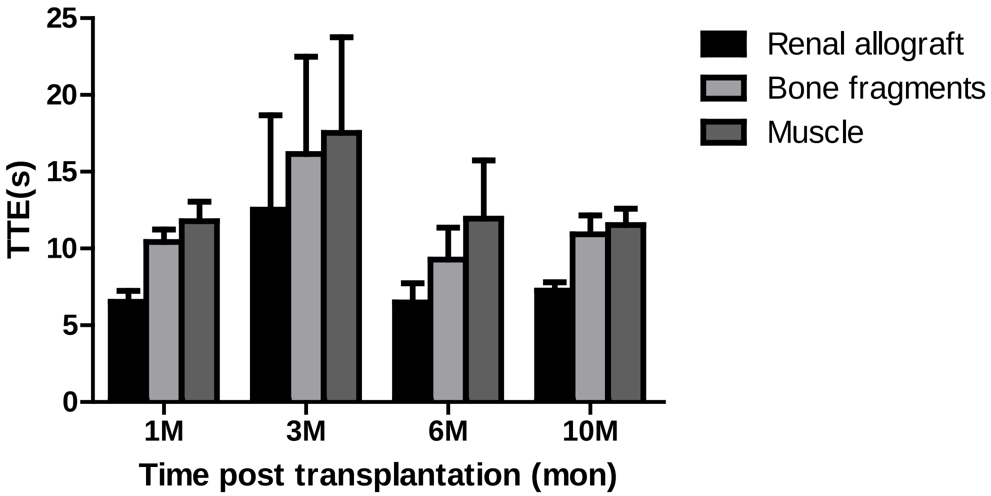

Figure 1B

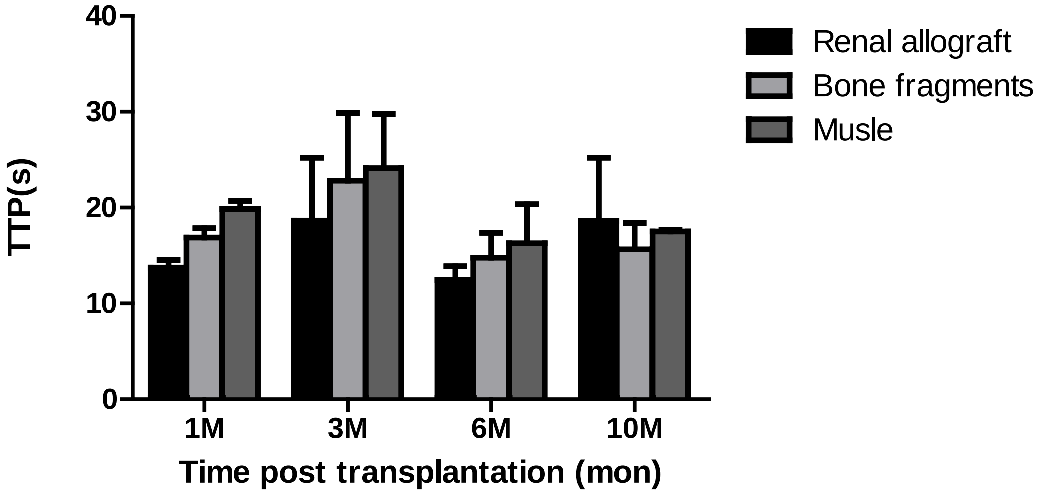

Figure 1C

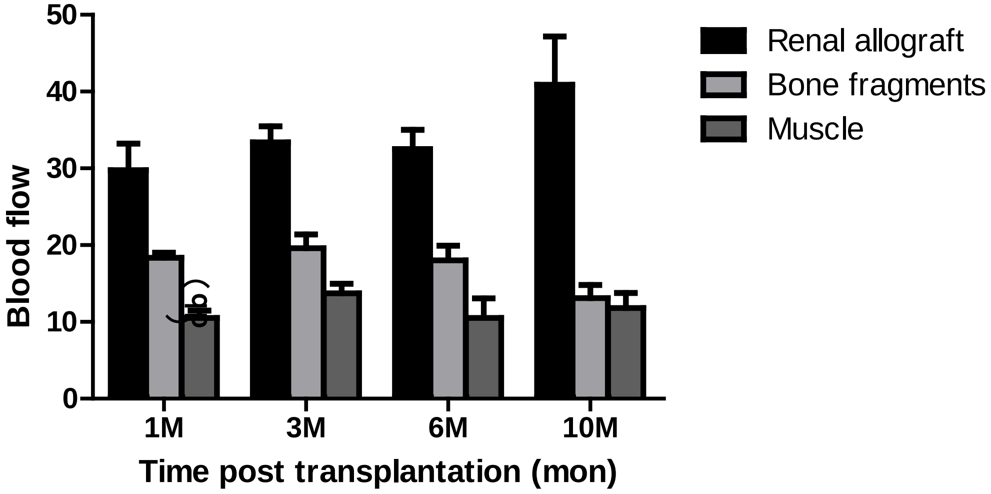
50
40
30
20
10
0
Blood flow
1M
3M
6M
10M
Time post transplantation (mon)
Renal allograft
Bone fragments
Muscle
